# Characterization of Gut Bacteria in *Blepharipa tibialis* (Diptera: Tachinidae) Larvae Parasitizing Different Developmental Stages of *Antheraea pernyi*

**DOI:** 10.3390/insects17050519

**Published:** 2026-05-19

**Authors:** Peng Hou, Li Liu, Ding Yang, Chuntian Zhang

**Affiliations:** 1 College of Life Science and Bioengineering, Shenyang University, Shenyang 110044, China; hp_0829@syu.edu.cn (P.H.); liujiali0229@163.com (L.L.); 2 College of Plant Protection, China Agricultural University, Beijing 110193, China; dyangcau@126.com; 3 College of Life Science, Shenyang Normal University, Shenyang 110034, China

**Keywords:** *Blepharipa tibialis*, gut bacteria, parasitoids, tachinids, *Antheraea pernyi*

## Abstract

*Blepharipa tibialis* (Diptera: Tachinidae) is a typical parasitoid fly. It spends most of its lifespan inside its host (*Antheraea pernyi* larvae) and relies heavily on host nutrition for survival. Whether the gut bacteria characteristics of *B. tibialis* larvae are associated with host development during the parasitoid process remains unclear. In this study, we used high-throughput sequencing to conduct the first systematic investigation of the gut bacteria of a parasitoid fly, comprehensively revealing the composition, structure, diversity, specificity, and potential functions in *B. tibialis* larvae parasitizing different host developmental stages. Results show that *B. tibialis* larval gut bacterial species were highly abundant, with a total of 24 phyla, 41 classes, 84 orders, 127 families, and 194 genera annotated. Although the number of bacterial species in *B. tibialis* larvae parasitizing different host stages (3rd, 4th, and 5th instar) differed significantly, the community structures were similar, suggesting that host physiological changes and dynamic alterations in the internal microenvironment may drive changes in the larval gut bacteria. Different host developmental stages may alter the gut bacterial composition of *B. tibialis* larvae, but bacterial functional stability is largely maintained. PICRUSt2 functional prediction indicated that the gut bacterial community may play an important role during the parasitoid process of *B. tibialis* larvae. This study provides an important basis for research on gut bacteria in tachinid flies (Diptera), supplements gut microbiota data for this group, and offers references for exploring the interaction mechanisms of gut microbiota in parasitoid systems, as well as for developing green control strategies against *A. pernyi* pests.

## Simple Summary

Analyzing the characteristics of insect gut microbiota is a critical perspective for exploring insect–bacteria interactions. Tachinidae (Diptera) are important biological control agents, all of which are parasitoids, playing key roles in stabilizing phytophagous insect populations and conserving biodiversity. However, the gut bacteria of parasitoid flies remain poorly explored. Here, using 16S rRNA gene sequencing, we systematically characterized the gut bacteria of *Blepharipa tibialis* (Diptera: Tachinidae) larvae parasitizing different developmental stages (3rd, 4th, and 5th instar) of the host *Antheraea pernyi* (Lepidoptera: Saturniidae) larvae, revealing the gut bacterial features of this tachinid parasitoid. Our findings report the first data on the gut bacteria of Tachinidae, provide a novel perspective for developing green control strategies against *A. pernyi* pests, and establish a critical foundation for future research on gut microbiota within host–parasitoid systems.

## 1. Introduction

Parasitoid insects are important biological control agents in nature, playing a crucial role in maintaining ecosystem stability and preserving biodiversity [1,2]. Parasitoid insects differ from predatory insects. They typically do not kill the host directly. Instead, they regulate the host’s growth and development, nutritional metabolism, and immunity to ensure the survival of their offspring, ultimately killing the host. Tachinidae is the second most species-rich family within the order Diptera and represents the most diverse and ecologically significant group of parasitoid insects among all taxa excluding hymenopteran parasitoid wasps [3,4,5]. All tachinid species exhibit a parasitoid lifestyle, with approximately 9000 described species worldwide [2,6]. Tachinid flies are strong fliers and have a broad host range. Their larvae parasitize phytophagous larvae of Lepidoptera, Coleoptera, Orthoptera, sawflies (Hymenoptera: Symphyta), and true bugs (Hemiptera: Heteroptera). These insects damage agricultural, forestry, forage, and fruit crops (e.g., corn borers, meadow moths, armyworms, cotton bollworms, pine caterpillars, gypsy moths, scarab beetles) [7,8]. Additionally, their larvae parasitize economically valuable insects such as the tussah silkworm (*Antheraea pernyi*) and the domestic silkworm (*Bombyx mori*) [8,9]. Briefly, tachinid flies are representative and important natural enemy insects. They regulate phytophagous insect populations and serve as ideal model organisms for studying host ecological adaptation and host–parasitoid interactions.

The genus *Blepharipa* belongs to the tribe Goniini (subfamily Exoristinae, family Tachinidae). Species of this genus lay microtype eggs and employ an indirect oviposition strategy, i.e., depositing eggs near the host. Indirect oviposition strategies are considerably more common in tachinid flies than in parasitoid hymenopterans, with approximately 40% of Palaearctic tachinid species using such strategies [7,10]. Worldwide, 28 species of *Blepharipa* are known, of which 16 occur in China [6,11]. Among these, *Blepharipa tibialis* is a typical representative and dominant species, and is also a pest that damages *A. pernyi*, causing significant economic losses in severe years [12,13,14].

**Figure 1:**
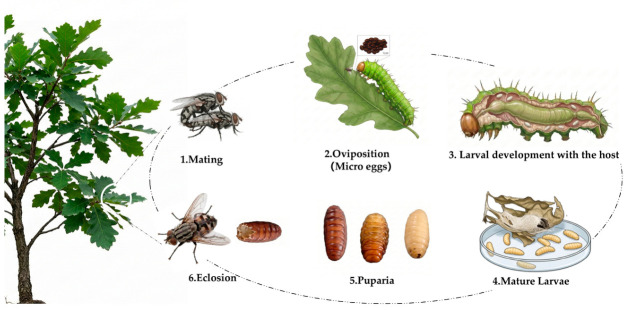
Scheme of the life cycle of *B. tibialis* parasitizing *A. pernyi* larvae.

*A. pernyi* (Lepidoptera: Saturniidae, Antheraea) is an economically important resource insect, and each stage of its life cycle has significant utilization value [15,16]. *A. pernyi* is the primary host of the parasitoid tachinid fly *B. tibialis*. Statistical data show that the parasitism rate is generally around 30% in normal years and can exceed 70% in severe years [13]. In Liaoning Province, China, *B. tibialis* has one generation per year, overwintering as pupae in the soil and emerging as adults in early May. Its oviposition peak coincides with the larval development period of the spring generation of *A. pernyi*. The offspring of *B. tibialis* obtain nutrition directly from the host throughout their development. Adult female flies deposit micro-eggs on the foliage of host plants. These eggs are subsequently ingested by *A. pernyi* larvae. Under the action of intestinal fluid, the eggs hatch into 1st-instar larvae, which migrate briefly, penetrate the intestinal wall, and form parasitic cysts beneath the larval cuticle of the host [14,17]. Larvae of all five instars of *A. pernyi* can be parasitized, with the 3rd instars being the most susceptible [17]. Due to the parasitoid habit of tachinid flies, the host does not die immediately after being parasitized by *B. tibialis*. Most parasitized hosts show no visible changes and are not easily detected. However, parasitism eventually causes *A. pernyi* larvae to die prematurely or fail to pupate, and even if pupation occurs, the host dies inside the cocoon. Ultimately, the mature 3rd-instar larvae of *B. tibialis* exit the cocoon, burrow into the soil, and overwinter as pupae, awaiting emergence the following year [12,17,18] (Figure 1).

The primary current method for controlling larval parasitism of *A. pernyi* by *B. tibialis* involves the application of organophosphorus agents. These agents inhibit acetylcholinesterase activity in the fly larvae, leading to excessive accumulation of the neurotransmitter acetylcholine, which disrupts neural transmission and ultimately causes paralysis and death of the larvae [19]. However, organophosphorus agents exhibit non-target toxicity, harming *A. pernyi* larvae themselves and posing chronic risks to the human nervous system. Studies have shown that at non-toxic concentrations for *A. pernyi* larvae, the control efficacy of these agents against the parasitoid fly is limited. Although increasing the concentration can enhance control efficacy, it often results in a significant decrease in the cocooning rate, making it difficult to achieve a balance between effective parasite control and cocoon yield. Numerous studies have confirmed that integrating gut bacterial characterization with insect growth, development, and reproduction is a critical perspective for exploring host–bacteria interaction mechanisms and an indispensable component for developing innovative pest management strategies [20,21,22,23].

To date, research on the gut bacteria of parasitoid insects remains limited. Whether the characteristics of gut bacteria in parasitoid flies are associated with host development during the parasitoid process remains unclear. Existing studies have primarily focused on hymenopteran parasitoid wasps [24,25,26,27], whereas no reports are available on the gut bacteria of dipteran tachinid flies. For example, one study reported that parasitism by *Cotesia vestalis* significantly altered the midgut bacterial community structure of *Plutella xylostella* larvae [28]. *Leptopilina boulardi* successfully developed in *Drosophila melanogaster* hosts that possessed gut bacteria but failed to develop in axenic hosts [29].

Given the economic importance of *A. pernyi* and the lack of gut bacterial data for tachinid flies, this study aims to characterize the gut bacteria of *B. tibialis* larvae parasitizing hosts at different developmental stages (3rd, 4th, and 5th instar). This study provides a foundation for future research on the bacteria of dipteran tachinids, supplements gut microbiota data for this group, and offers a reference for exploring the interaction mechanisms of gut microbiota in parasitoid systems, as well as for developing green control strategies against pests of *A. pernyi*.

## 2. Materials and Methods

### 2.1. Host Larvae Rearing and B. tibialis Larvae Collection

The host larvae (*A. pernyi*) used in the experiment were obtained from the ‘Ji Qing’ bivoltine strain provided by the Jilin Provincial Institute of Sericulture Science. Larvae were reared from eggs in April 2025 at a Mongolian oak (*Quercus mongolica*) silkworm farm in Qingliangshan Town, Xiuyan County, Anshan City, Liaoning Province, China (40°35′26.75″ N, 123°40′31.80″ E; altitude 298 m). Environmental parameters were as follows: average temperature 22–25 °C, relative humidity 80–85%, and photoperiod 14L:10D. The duration from egg to the first day of the 5th instar was 45 ± 2 days (mean ± SD). Specifically, the duration from egg to the 2nd instar was 8 ± 2 days, from the second to the 3rd instar was 8 ± 2 days, from the 3rd to the 4th instar was 12 ± 2 days, and from the 4th to the 5th instar was 16 ± 2 days (mean ± SD).

To minimize the interference of pesticides with oviposition by adult *B. tibialis* parasitizing *A. pernyi* larvae, no pesticides were sprayed on the experimental area. Potentially parasitized 3rd, 4th, and 5th instar larvae were collected separately (100 larvae per instar) and transported to the Key Laboratory of Urban Pest Control and Ecological Safety in Liaoning Province, where they were reared until the emergence of mature 3rd-instar *B. tibialis* larvae. During laboratory rearing, environmental parameters and food sources were strictly controlled to maintain consistency with those in the silkworm farm rearing field.

The parasitism rates of the collected *A. pernyi* larvae were 32% for the 3rd instar, 21% for the 4th instar, and 27% for the 5th instar. The number of 3rd-instar *B. tibialis* larvae emerging per host larva ranged from 4 to 6. A total of 412 3rd-instar *B. tibialis* larvae were collected, comprising 168 individuals that had parasitized the 3rd-instar host, 116 from the 4th-instar, and 133 from the 5th-instar. These samples were designated as BT3, BT4, and BT5, where BT3 denotes the 3rd-instar *B. tibialis* larvae parasitizing 3rd-instar host larvae, BT4 those from the 4th-instar hosts, and BT5 those from the 5th-instar hosts.

### 2.2. Gut Isolation and Total DNA Extraction

Sixty larvae were randomly selected from each of the groups BT3, BT4, and BT5. The larvae were rinsed with 4% sodium chloride solution pre-chilled to 4 °C until no visible foreign matter was observed, followed by surface disinfection with 75% ethanol for 60 s. This disinfection step was repeated three times, and then the larvae were rinsed three times with sterile water. These procedures were performed to minimize potential interference from surface contaminants and microorganisms on subsequent research. After cleaning, the larvae from each group were aseptically dissected on a clean bench to obtain gut samples. The gut tissues were rinsed three times with 0.9% NaCl solution until no visible contents remained, then transferred to 1.5 mL centrifuge tubes, rapidly frozen in liquid nitrogen, and stored at −80 °C for later use. Three biological replicates were set up for each group (guts from 20 randomly selected larvae were pooled into one biological replicate). Finally, 3rd-instar larval gut samples of the *B. tibialis* larvae were obtained for the three groups, each containing three biological replicates, designated as BT3_1, BT3_2, BT3_3; BT4_1, BT4_2, BT4_3; and BT5_1, BT5_2, BT5_3, respectively.

Frozen samples of *B. tibialis* larvae were homogenized using sterile grinding rods. Following the manufacturer’s instructions, total DNA was extracted from the larval gut tissues of each group using the DNeasy DNA Extraction Kit (TIANGEN, Beijing, China) for insect *COI* gene sequencing, to verify whether all sampled larvae were the tachinid fly *B. tibialis*. The specific procedures and methods followed the standard protocols of Hou et al. [30]. Total gut bacterial DNA was extracted from each group using the E.Z.N.A.^®^ Soil DNA Kit (Omega Bio-tek, Norcross, GA, USA). DNA contamination and degradation were assessed by electrophoresis on a 1% agarose gel. DNA purity was evaluated by measuring the OD260/OD280 ratio using a NanoDrop 2000 spectrophotometer (Thermo Scientific, Wilmington, NC, USA), and its concentration and integrity were determined using a Qubit fluorometer (Invitrogen, Carlsbad, CA, USA) and an Agilent 2100 bioanalyzer (Agilent, Santa Clara, CA, USA).

### 2.3. PCR Amplification and Sequencing

PCR amplification was performed using the forward primer 338F (5′-ACTCCTACGGGAGGCAGCAG-3′) and reverse primer 806R (5′-GGACTACHVGGGTWTCTAAT-3′), which target the V3–V4 hypervariable region of the bacterial 16S rRNA genes. A PCR blank without a DNA template (using ddH_2_O instead) was included to check for contamination during amplification. PCR mixture was prepared in a total volume of 20 µL, containing 1 µL of template DNA (10 ng/µL), 4 μL of 5× FastPfu Buffer, 2 μL of 2.5 mM dNTPs, 0.8 µL of each primer (5 µM), and 0.4 μL of FastPfu DNA Polymerase. PCR conditions were as follows: initial denaturation at 95 °C for 3 min; followed by 29 cycles of 95 °C for 30 s, 55 °C for 30 s, and 72 °C for 45 s; with a final extension at 72 °C for 10 min (PCR instrument: ABI GeneAmp^®^ 9700, Union City, CA, USA). PCR products were separated on a 2% agarose gel, and the target bands were excised and purified using the AxyPrep DNA Gel Extraction Kit (Axygen, Union City, CA, USA).

Purified products were quantified using a Qubit^®^ 4.0 Fluorometer (Thermo Fisher Scientific, USA). Library preparation was performed using the NEBNext Ultra II DNA Library Prep Kit (New England Biolabs, Ipswich, MA, USA), and sequencing was carried out on the Illumina MiSeq PE 300 platform (Shanghai Majorbio Technology, Shanghai, China).

### 2.4. Sequencing Data Processing

Quality control of paired-end raw sequencing data was performed using fastp (version 0.23.4; https://github.com/OpenGene/fastp, accessed on 1 September 2025), and read merging was conducted using FLASH (version 1.2.11; https://ccb.jhu.edu/software/FLASH/index.shtml, accessed on 1 September 2025). The specific parameters and workflow followed the quality control and merging procedures described by Hou et al. [31]. Denoising and chimera removal were performed using the DADA2 plugin implemented in QIIME 2 software (version 2024.2; https://qiime2.org, accessed on 4 September 2025), yielding amplicon sequence variants (ASVs). Taxonomic annotation of ASVs was performed using the Naive Bayes classifier in QIIME 2 against the SILVA 16S rRNA gene database (version 138; https://www.arb-silva.de/, accessed on 5 September 2025) with a confidence threshold of 0.7, and ASVs annotated as chloroplasts or mitochondria were removed from the feature table. Based on the ASV data, the functional potential of gut bacteria was predicted using PICRUSt2 (version 2.2.0; https://github.com/picrust/picrust2/, accessed on 5 September 2025) against the KEGG database.

Based on the NCBI database, the *COI* gene sequence of the insect samples was aligned with the existing references *COI* gene sequence of *B. tibialis* (Accession No. MG520274; EU433559), showing 100% identity.

The raw sequencing data of 16S rRNA genes were deposited in the Sequence Read Archive (SRA) under the following accession numbers: for BT3, SRR38075398, SRR38075397, and SRR38075396; for BT4, SRR38075395, SRR38075394, and SRR38075393; for BT5, SRR38075392, SRR38075391, and SRR38075390. The *COI* data of all insect samples are available under NCBI GenBank No. PZ278983.

### 2.5. Bioinformatic Analysis

Rarefaction curves were plotted based on ASVs to assess sequencing coverage and verify data reliability. Alpha diversity indices, including Chao1, ACE, Shannon, and Simpson indices, were calculated using Mothur (version 1.30.2). The Kruskal–Wallis test was used to compare diversity indices among groups. The false discovery rate (FDR) was applied to adjust *p*-values for multiple testing, followed by Dunn’s post hoc test with FDR correction, and results were visualized accordingly. Beta diversity analysis was performed using non-metric multidimensional scaling (NMDS) based on the Bray–Curtis dissimilarity matrix to visualize the overall separation among sample groups. Analysis of similarities (ANOSIM) with 999 permutations was applied to test the significance of differences in community structure between groups. Linear discriminant analysis effect size (LEfSe) (http://huttenhower.sph.harvard.edu/galaxy/; accessed on 5 September 2025) was used to identify taxa with significant differences that most likely explain bacterial variations (LDA score > 3, *p* < 0.05). Gene functions of the gut bacteria were predicted using PICRUSt2 (version 2.5.2) against the KEGG database (release 2024), and one-way analysis of variance (ANOVA) was used to compare the relative abundances of predicted KEGG pathways among groups.

## 3. Results

### 3.1. General Sequencing Data Results

**Figure 2:**
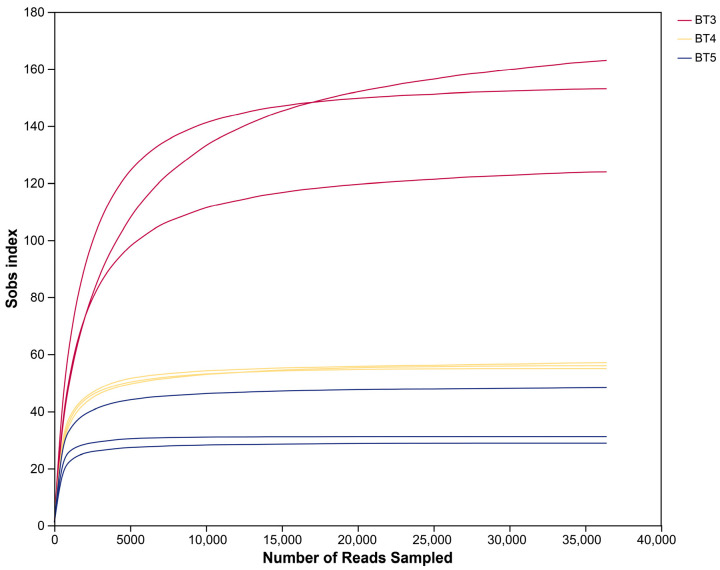
Rarefaction curve of gut bacteria in *B. tibialis* Larvae.

High-throughput sequencing of the V3–V4 region of the 16S rRNA gene was performed on samples BT3, BT4, and BT5, each in three biological replicates. A total of 948,540 raw reads were obtained. After paired-end merging, quality trimming, and filtering, 400,395 high-quality sequences were obtained, with an average read length per sample ranging from 406 to 426 bp. Following denoising based on single-nucleotide differences and removal of sequences assigned to chloroplasts or mitochondria, 485 ASVs were identified across all samples. The number of ASVs per biological replicate ranged from 26 to 163. When combining the three replicates per group, the total ASVs were 344 (BT3), 138 (BT4), and 76 (BT5). Notably, the ASVs in BT3 was higher than those in BT4 and BT5. Rarefaction curve (Figure 2) shows that as sequencing depth increases, the sobs index in each sample group gradually stabilizes and eventually reaches a plateau. Taxonomic annotation revealed a total of 24 phyla, 41 classes, 84 orders, 127 families, and 194 genera (Table 1).

### 3.2. Composition and Structure of Gut Bacteria in B. tibialis Larvae

**Figure 3:**
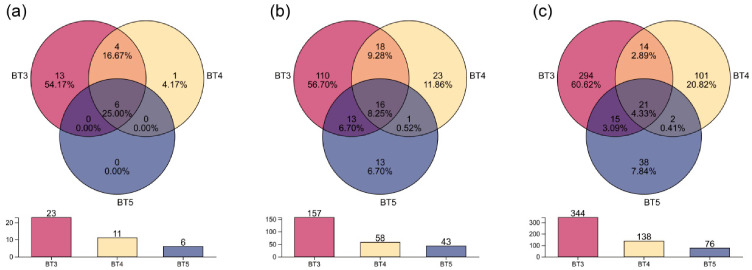
Venn diagrams and bar charts of gut bacteria in *B. tibialis* larvae across samples. (**a**) At the phylum level. (**b**) At the genus level. (**c**) At the ASV level.

A combination of Venn diagrams and bar charts visualized the compositional characteristics of BT3, BT4, and BT5 at the phylum and genus taxonomic annotation levels, as well as at the ASV level. At the phylum level, BT3 exhibited the highest number of unique phyla (13), accounting for 54.17% of all annotated phyla across samples, whereas BT4 and BT5 had 1 and 0 unique phyla, respectively. The number of phyla shared among all three groups was 6 (25.00%). Exclusively between BT3 and BT4 (i.e., excluding the three-group shared phyla), 4 phyla (16.67%) were shared. No phyla were shared exclusively between BT3 and BT5 or between BT4 and BT5 (Figure 3a). The number of genera shared among all three groups was 16 (8.25%). Excluding the three-group shared genera, BT3 and BT4 shared 18 genera, BT3 and BT5 shared 13 genera, whereas BT4 and BT5 shared only 1 genus (Figure 3b). At the ASV level, BT3 had 294 unique ASVs (60.62% of total ASVs), substantially higher than BT4 (101, 20.82%) and BT5 (38, 7.84%). Moreover, the number of shared ASVs among groups was generally low: 21 ASVs (4.33%) were shared by all three groups; 14 ASVs (2.89%) were shared exclusively by BT3 and BT4; 15 ASVs (3.09%) were shared exclusively by BT3 and BT5; and only 2 ASVs (0.41%) were shared exclusively by BT4 and BT5 (all excluding the three-group shared ASVs) (Figure 3c). The bar charts further illustrated the counts at each taxonomic level and ASV level across groups: BT3 consistently had higher total numbers (i.e., the sum of unique and shared taxa within each group) than BT4 and BT5, with 23, 157, and 344 at the phylum, genus, and ASV levels, respectively, whereas BT5 consistently had the lowest totals, with 6, 43, and 76, respectively.

**Figure 4:**
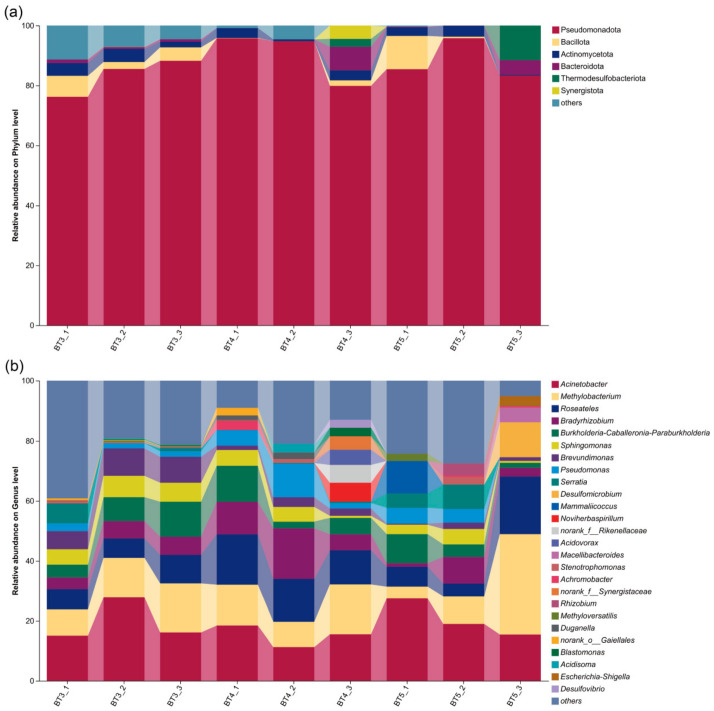
Composition of gut bacteria in *B. tibialis* larvae across samples at different taxonomic levels. (**a**) At the phylum level. (**b**) At the genus level.

Based on the analysis of bacterial community relative abundance using ASVs, the gut bacteria of *B. tibialis* larvae were dominated at the phylum level by Pseudomonadota (87.24%), followed by Bacillota (3.02%) and Actinomycetota (2.75%), also including Bacteroidota (1.72%), Thermodesulfobacteriota (1.57%), and Chloroflexota (with relative abundances below 1% classified as others), although their relative abundances varied among the three experimental groups. Pseudomonadota was the predominant phylum across all groups, exhibiting the highest relative abundance in each. Thermodesulfobacteriota showed a higher relative abundance in the BT5 group than in the other two groups. Synergistota was detected in both the BT3 and BT4 groups. However, its abundance was low in the BT3 group (merged into others), whereas it reached a relative abundance of 1.47% in the BT4 group (Figure 4a).

At the genus level, BT3, BT4, and BT5 groups shared *Acinetobacter* and *Methylobacterium* as the predominant genera. In the BT3 group, the relative abundances of *Burkholderia–Caballeronia–Paraburkholderia* (8.00%) and *Brevundimonas* (7.96%) were also relatively high. In the BT4 group, the abundances of *Roseateles* (14.18%) and *Bradyrhizobium* (11.01%) were higher than those in the BT3 group (7.85% and 5.21%) and BT5 group (10.02% and 4.34%). The BT5 group exhibited specific enrichment of *Desulfomicrobium* and *Mammaliicoccus* (Figure 4b).

**Figure 5:**
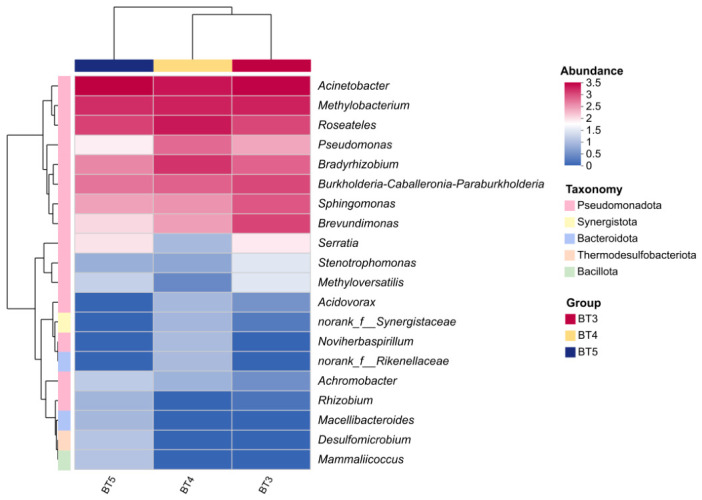
Clustered heatmap of top 20 abundant gut bacterial communities in *B. tibialis* larvae across samples. Note: Rows and columns represent the samples and the dominant taxon, and the right side of the figure is the value represented by the color gradient.

Further species abundance clustering heatmap analysis of the top 20 genera revealed that, at the horizontal sample clustering, the BT3 and BT4 groups first clustered together, and then clustered with the BT5 group. This clustering pattern indicates that the BT3 and BT4 groups share certain similarities in community structure, while exhibiting inter-group differences with the BT5 group. In the vertical genus clustering, genera with similar abundance variation patterns were clustered together. In the BT3, BT4, and BT5 groups, Pseudomonas was the dominant taxon, while *Methylobacterium* and *Roseobacter* first clustered together, and then clustered with *Acinetobacter* (Figure 5).

### 3.3. Diversity Characteristics of Gut Bacteria in B. tibialis Larvae

In this study, the diversity characteristics of the gut bacteria of *B. tibialis* larvae (BT3/BT4/BT5) parasitizing different developmental stages of the host were systematically analyzed at the ASVs level by comparing Alpha diversity indices (ACE, Chao1, Shannon, and Simpson indices) (Table 2). In terms of species richness, the BT3 group exhibited the highest Sobs, ACE, and Chao1 indices, followed by the BT4 group, with the BT5 group showing the lowest values. This indicates that the gut bacteria richness of *B. tibialis* larvae parasitizing the 3rd-instar stage of the host is the highest and considerably greater than that of the other two groups, suggesting a more complex gut bacterial ecosystem in the BT3 group. Furthermore, the Coverage values for all three groups were ≥0.998, indicating that the current sequencing depth covered more than 99.8% of the bacterial species. The data are highly reliable, and the observed differences in richness are not due to insufficient sequencing but rather objectively reflect the actual bacterial community composition in the samples.

**Figure 6:**
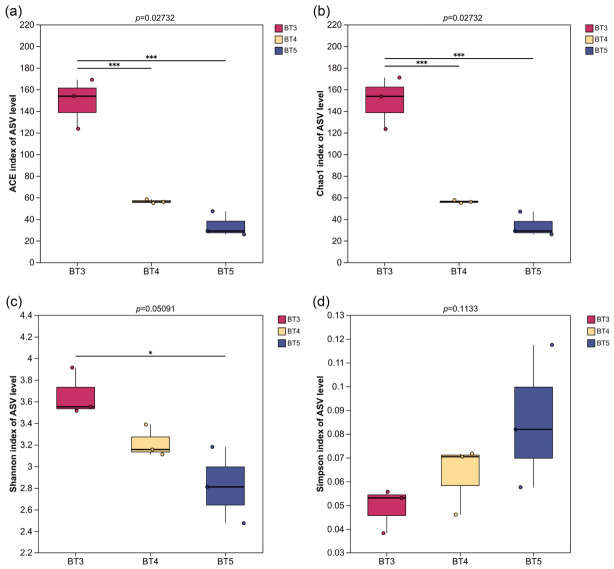
Kruskal–Wallis tests of Alpha diversity indices in *B. tibialis* larvae across samples. (**a**,**b**) Boxplot of species richness (number of ASVs) assessed by the ACE and Chao1 indices. (**c**,**d**) Boxplot of bacterial community diversity assessed by the Shannon and Simpson indices. Note: * indicates significant differences (*p* < 0.05), *** indicates extremely significant differences (*p* < 0.001).

In terms of species diversity, the Shannon index followed the order BT3 > BT4 > BT5, while the Simpson index followed BT3 < BT4 < BT5. Both indices consistently indicated that the BT3 group had the highest bacterial diversity, followed by the BT4 group, and the BT5 group had the lowest. Notably, BT5 not only exhibited a lower number of bacterial taxa but also showed a relatively high Simpson index (0.09), suggesting a more pronounced dominance of certain species and lower community evenness. Furthermore, the relatively large standard deviations of various indices in the BT3 group (e.g., Sobs ±21.38, Chao ±24.10) indicate some degree of variation among biological replicates, which may reflect that the gut bacteria of this group are more sensitive to microenvironmental changes or that individual physiological states differ. Kruskal–Wallis tests performed on the alpha diversity indices across groups revealed statistically significant differences in bacterial community richness among the groups, and these significant differences held in all pairwise comparisons. In contrast, no statistically significant differences were found in bacterial community diversity among the groups (Figure 6).

**Figure 7:**
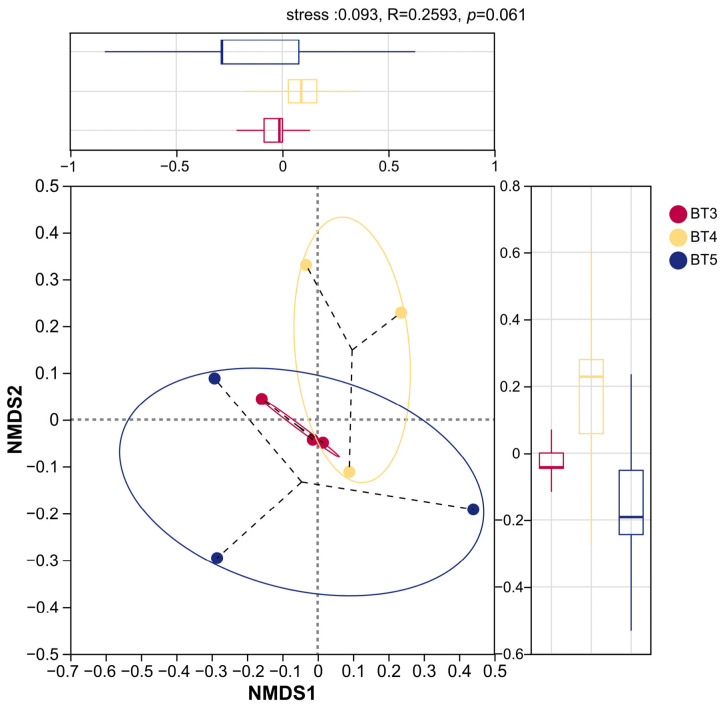
NMDS plot showing the beta diversity characteristics in *B. tibialis* larvae across samples.

NMDS analysis based on the Bray–Curtis algorithm was further conducted to explore the beta diversity characteristics of the bacterial community structures among the BT3, BT4, and BT5 groups (Figure 7). As shown in the figure, the stress value was 0.093 (<0.1), indicating a good fit of the results, which reliably reflect the beta diversity relationships among the samples. The closer the sample points are in the plot, the more similar the bacterial community structures. Results show that the sample points of the BT3 group were relatively concentrated, indicating good within-group reproducibility. The sample points of the BT4 group were mainly distributed in the first and fourth quadrants, forming a relatively clustered community distribution, whereas the BT5 group exhibited a more dispersed distribution, suggesting differences in bacterial community structures within the BT5 group. There was partial spatial overlap between the BT3 and BT4 groups, with relatively close distances between sample points, indicating a certain degree of similarity in their bacterial community structures. Although the BT3 and BT5 groups showed obvious spatial overlap, the distances between sample points were relatively large, indicating certain differences in their bacterial community structures. In summary, the bacterial community structures of the BT3, BT4, and BT5 groups exhibited a trend of separation and differential characteristics. However, the ANOSIM test (R = 0.2593, *p* = 0.061) indicated that the overall structure of the gut bacterial communities among the three groups did not reach statistical significance.

### 3.4. Differences and Specificity of Gut Bacteria in B. tibialis Larvae

**Figure 8:**
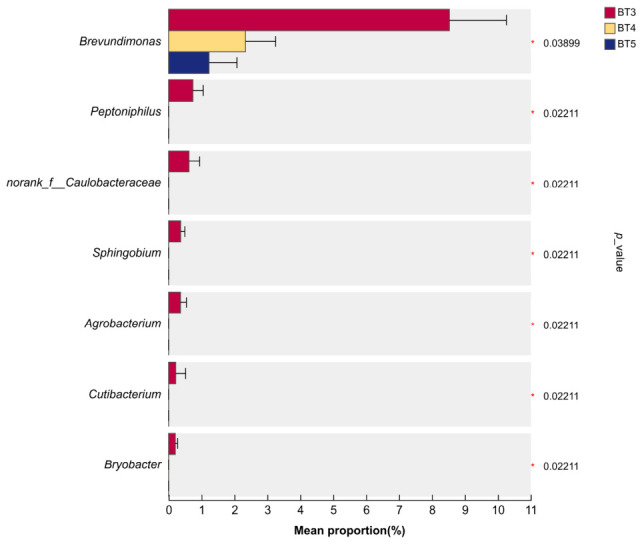
Significance analysis of the top 20 bacterial genera in *B. tibialis* larvae across samples. Note: * indicates significant differences (*p* < 0.05).

Kruskal–Wallis test with Dunn’s post hoc correction identified seven bacterial genera with significantly different relative abundances among the BT3, BT4 and BT5 groups: *Brevundimonas, Peptoniphilus*, norank_f__Caulobacteraceae, *Sphingobium*, *Agrobacterium*, *Cutibacterium*, and *Bryobacter* (Figure 8). Across the three groups, the relative abundance of *Brevundimonas* significantly decreased (BT3 > BT4 > BT5). In contrast, the other six genera had relative abundances close to zero (<0.001%) in BT4 and BT5 groups, which were significantly lower than those in BT3.

**Figure 9:**
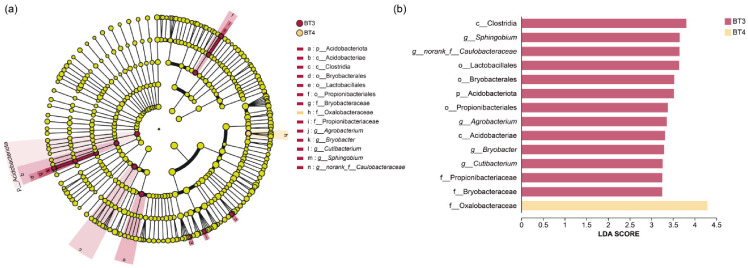
LEfSe analysis of gut bacteria in *B. tibialis* larvae across samples. (**a**) Cladogram of bacterial taxa enriched across samples. (**b**) LDA discriminates histogram. Note: Phyla (p), Class (c), Order (o), Family (f), Genus (g).

LEfSe was performed to identify specific bacterial biomarkers at phylum, class, order, family, and genus levels, with an LDA threshold > 3. The results show that the BT3 group had the highest number of significantly different bacterial taxa, including 1 phylum, 2 classes, 3 orders, 3 families, and 4 genera. In contrast, the BT4 group had only one significantly different taxon at the family level, and the BT5 group had none (Figure 9a). As shown in the LDA bar plot (Figure 9b), the class Clostridia showed the highest LDA score in the BT3 group, followed by the genus *Sphingobium*, the unclassified family Caulobacteraceae, the order Lactobacillales, the order Bryobacterales, the phylum Acidobacteriota, the order Propionibacteriales, the genus *Agrobacterium*, the class Acidobacteriae, the genus *Bryobacter*, the genus *Cutibacterium*, the family Propionibacteriaceae, and the family Bryobacteraceae. All these taxa were identified as specific biomarkers of the BT3 group. For the BT4 group, only the family Oxalobacteraceae was a specific biomarker.

### 3.5. Functional Prediction of Gut Bacteria in B. tibialis Larvae

**Figure 10:**
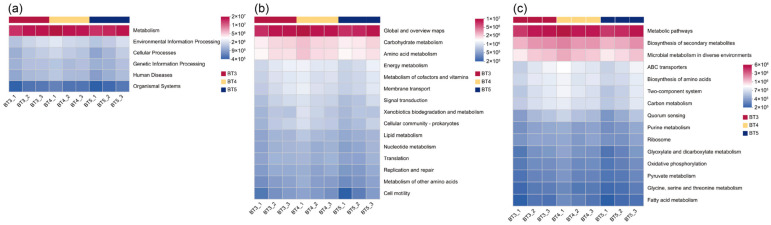
KEGG pathway annotations of gut bacteria functional potential in *B. tibialis* larvae across samples. (**a**) KEGG primary pathway annotation level. (**b**) KEGG secondary pathway annotation level. (**c**) KEGG third pathway annotation level. Note: The color gradient of the blocks was utilized to illustrate changes in functional abundance across different groups. A higher value corresponds to greater functional abundance.

**Figure 11:**
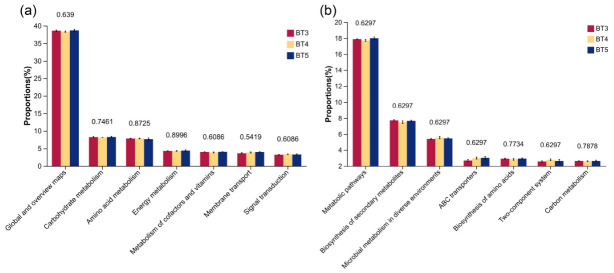
ANOVA-based differential analysis of predicted gene functional potential of gut bacteria in *B. tibialis* Larvae across samples. (**a**) KEGG secondary pathway annotation level. (**b**) KEGG third pathway annotation level.

Functional potential of the gut bacteria of *B. tibialis* larvae was predicted using PICRUSt2 against the KEGG database. At the KEGG pathway level 1, the abundance of the Metabolism pathway was highest across all three groups (BT3, BT4, and BT5), followed by Environmental Information Processing and Cellular Processes, while Organismal Systems showed the lowest abundance in all groups (Figure 10a). At level 2, Global and overview maps exhibited the highest abundance, followed by Carbohydrate Metabolism and Amino Acid Metabolism (Figure 10b). At level 3, the Metabolic Pathways, Biosynthesis of Secondary Metabolites, and Microbial Metabolism in Diverse Environments showed relatively high abundance across all three groups (Figure 10c). ANOVA revealed no significant differences in the predicted functional pathway abundances among the BT3, BT4, and BT5 groups at any KEGG level (*p* > 0.05). However, certain numerical trends in relative abundance were observed. Specifically, the BT5 group tended to have higher abundance in Global and overview maps, Carbohydrate Metabolism, and Energy Metabolism compared with the other two groups. In contrast, the BT3 group showed a higher proportion of Amino Acid Metabolism, while the BT4 group exhibited a relatively higher abundance of Signal Transduction (Figure 11a). At level 3, Metabolic Pathways were the most abundant pathway, with its abundance in the BT5 group being numerically higher than in the BT3 and BT4 groups. The Biosynthesis of Secondary Metabolites pathway was most abundant in the BT3 group, whereas the Microbial Metabolism in Diverse Environments pathway was most abundant in the BT4 group (Figure 11b).

## 4. Discussion

Gut bacteria of parasitoid insects not only participate in their own nutritional metabolism and immune regulation but also influence the gut bacteria composition of their parasitoid hosts, thereby modulating the host’s physiological functions and ecological adaptation [26,27,29,32]. Parasitoid flies are important biological control agents regulating lepidopteran insect populations and are of significant research value. *B. tibialis* is a typical parasitoid fly, with most of its life cycle spent inside the host, relying heavily on the host’s nutrients for survival. Gut bacteria of *B. tibialis* play important roles in the growth and development of its larval stage. The composition and structure of its gut bacteria may reflect the adaptation of the parasitoid process to the host microenvironment and may also exert potential functions in the host’s nutritional metabolism, immune regulation, and interactions with the host.

Through high-throughput sequencing of the V3–V4 region of the 16S rRNA gene from the samples, a total of 24 phyla, 41 classes, 84 orders, 127 families, and 194 genera of gut bacterial taxa associated with *B. tibialis* larvae were annotated, indicating a highly diverse gut bacterial community. At the phylum level, the gut bacteria of *B. tibialis* larvae parasitizing 3rd-instar *A. pernyi* larvae exhibited 13 unique phyla and 11 shared phyla compared with the other two groups. However, the composition of dominant phyla remained relatively stable across the three sample groups, primarily consisting of Pseudomonadota, Bacillota, and Actinomycetota. Among these, Pseudomonadota was the dominant phylum with high abundance, a pattern consistent with the general characteristics of gut bacteria in insects of Lepidoptera, Diptera, Hymenoptera, and others, and is closely associated with key physiological functions of the parasitoid-host system [27,33,34,35,36]. Furthermore, the major gut bacterial phyla of *B. tibialis* larvae were consistent with those previously reported in parasitoid wasps and matched earlier findings on the gut microbiota composition of its host, although differences were observed in the relative abundances of the major bacterial groups [16,28,31].

At the genus level, the dominant bacterial genera (with relative abundance >1%) in *B. tibialis* larvae are abundant, and mainly consist of aerobic and facultatively anaerobic bacteria, including *Acinetobacter*, *Methylobacterium*, *Brevundimonas*, *Roseobacter*, *Bradyrhizobium*, *Sphingomonas*, *Burkholderia–Caballeronia–Paraburkholderia*, *Sphingobium*, etc. Notably, a unique genus *Neoherbaspirillum* appeared in the BT4 group, and the strictly anaerobic genus *Desulfomicrobium* appeared in the BT5 group.

*Acinetobacter* is a facultative anaerobe capable of colonizing the microaerobic environment of the insect gut. Most strains secrete various extracellular enzymes that assist the host in breaking down proteins and polysaccharides. *Methylobacterium* is a methylotrophic bacterium that utilizes one-carbon compounds such as methanol and formaldehyde, which are abundant in the insect gut for metabolism [37]. In parasitoid environments, these bacteria may help *B. tibialis* larvae degrade the host’s fat body and detoxify plant secondary metabolites (e.g., tannins, alkaloids) accumulated in the host due to ingestion of plant leaves. The enrichment of *Brevundimonas*, *Burkholderia–Caballeronia–Paraburkholderia*, and *Roseateles* in the BT3 group may be associated with the greater need for plant polysaccharide degradation capacity and environmental adaptability during the early stage of the host *A*. *pernyi* larvae. Specifically, *Brevundimonas* possesses the ability to degrade complex polysaccharides, potentially assisting *B. tibialis* larvae in decomposing the nutrients from tissues or hemolymph of the host. *Burkholderia* has the potential to degrade organophosphorus pesticides and has been demonstrated to participate in the degradation of such pesticides in the bean bug *Riptortus pedestris* [38].

In the BT4 group, the abundances of *Roseateles* and *Bradyrhizobium* are higher than in the other two groups, which may be related to the increased efficiency of energy metabolism at this developmental stage of the host *A. pernyi* larvae. *Roseateles* is a photoheterotrophic bacterium that may assist *B. tibialis* larvae in generating ATP through anoxygenic photosynthesis under hypoxic conditions [36]. The specific enrichment of *Bradyrhizobium* may be linked to an increased demand for specific nitrogen sources within the host *A. pernyi* at this stage. This genus is primarily known for nitrogen fixation in legume root nodules, and its function in the insect gut warrants further exploration.

In the BT5 group, the specifically enriched *Desulfomicrobium* (belonging to the phylum Thermodesulfobacteriota) is a strictly anaerobic sulfate-reducing bacterium involved in energy metabolism and redox balance regulation under anoxic conditions. *Mammaliicoccus* (phylum Bacillota) possesses fermentative metabolic capacity, utilizing accumulated organic acids and sugars in the gut to produce short-chain fatty acids [39]. This shift may reflect a transition of the gut environment of *B. tibialis* larvae parasitizing late-stage *A. pernyi* hosts toward a more anaerobic and eutrophic state.

Further combined with alpha diversity indices and significance testing, this study revealed that although there was no statistically significant difference in the diversity of gut bacteria of *B. tibialis* larvae parasitizing different developmental stages of the hosts, there were significant differences in richness (richness: BT3 > BT4 > BT5). This indicates that while the abundance of gut bacteria in the parasitoid larvae across the three parasitoid stages varies considerably, the relative abundance structure of dominant bacteria remains relatively similar. This finding may be associated with the nutritional metabolism and immune regulation of the host at different developmental stages. Specifically, the entire larval development of *B. tibialis* occurs within the host, and the source of its gut bacteria is not primarily derived from external environmental intake but is rather influenced by internal selection. Some bacteria may be vertically transmitted via eggs (e.g., *Burkholderia–Caballeronia–Paraburkholderia*), whereas most are likely acquired by the parasitoid larvae from the host. The BT3 group comprises *B. tibialis* larvae parasitizing the 3rd instar of *A. pernyi* larvae, a host stage with vigorous nutritional metabolism, rapid immune development, high food intake, and rich gut microbiota [16,31]. The BT5 group comprises *B. tibialis* larvae parasitizing the 5th instar of *A. pernyi* larvae, a stage when the host ceases feeding and enters the prepupal phase, characterized by reduced food intake, simplified nutrient composition, a more developed immune system, and increased environmental selection pressure, which may lead to suppression or elimination of certain microbial populations within the host [16,31]. Therefore, the physiological changes and dynamic internal microenvironment of the host may be key factors driving the gut bacteria richness of *B. tibialis* larvae. Notably, the significant changes in richness coupled with non-significant changes in diversity may suggest that different developmental stages of the host alter the gut bacterial composition of *B. tibialis* larvae, while maintaining a functionally stable community structure, thereby ensuring the normal development of the parasitoid larvae.

NMDS analysis revealed no significant separation among the three groups (BT3/BT4/BT5) in overall community structure (*p*= 0.061), consistent with the observation that although certain bacteria differed in relative abundance, the overall community structure remained similar. BT3 samples were more concentrated, suggesting a potentially more stable gut bacteria structure, whereas BT5 samples were more dispersed, indicating increased inter-individual heterogeneity at this parasitic stage, which may be related to differences in host developmental status, parasitism duration, or microenvironmental conditions. Furthermore, BT3 and BT4 samples partially overlapped and showed closer distances, indicating higher community similarity between these two groups, while there was a trend of separation from BT5 samples. This finding largely agrees with the high similarity in bacterial structure between BT3 and BT4 revealed by genus-level clustering heatmap analysis.

LEfSe further identified bacterial biomarkers. BT3 group included *Sphingobium*, *Agrobacterium*, *Bryobacter*, *Cutibacterium*, etc. Among these, *Sphingobium* can degrade aromatic compounds and polycyclic aromatic hydrocarbons, potentially aiding *B. tibialis* larvae in metabolizing toxins in the parasitoid environment and enhancing their adaptability [40]. Oxalobacteraceae was the only biomarker in the BT4 group. This family utilizes oxalate as its sole carbon source and is often associated with plant material degradation or oxalate metabolism [41]. No distinct biomarkers were identified in the BT5 group, suggesting lower functional specialization of its gut bacteria, which may be related to reduced metabolic activity of the *A. pernyi* host.

PICRUSt2 gene function prediction analysis suggests that the gut bacteria may play an important role during parasitization of *B. tibialis* larvae. In all three groups of larval gut bacteria, Metabolism was the most abundant at Level 1 pathways. At Level 2, core pathways included Global and overview maps, Carbohydrate Metabolism, and Amino Acid Metabolism. At Level 3, the dominant pathways were Metabolic Pathways, Biosynthesis of Secondary Metabolites, and Microbial Metabolism in Diverse Environments. The functional pathway distribution was consistent across the three groups, indicating that the core metabolic functions of the gut bacteria in *B. tibialis* larvae may be conserved. This result aligns with functional predictions for gut bacteria in most insects. By participating in the metabolism of basic nutrients such as carbohydrates and amino acids, the gut microbiota compensates for deficiencies in the host digestive enzyme system and enhances nutrient utilization efficiency, reflecting a common functional trait of insect gut microorganisms. The high abundance of the secondary metabolite biosynthesis pathway suggests that the gut bacteria of *B. tibialis* larvae may possess the genetic potential to synthesize secondary metabolites, including antibiotics and signaling molecules. These metabolites may regulate the gut microecological balance of *B. tibialis* larvae and potentially interact with the host. No significant differences in functional prediction pathways were found among the three groups, which partially supports the hypothesis that while community composition exhibits some differentiation, core functions remain stable, reflecting functional redundancy. However, functional predictions based on the 16S rRNA gene V3–V4 region are indirect and cannot serve as direct evidence. Future validation using metagenomics, metabolomics, or in vitro experiments is required.

## 5. Conclusions

This study presents the first systematic investigation of the gut bacteria of parasitoid flies, revealing the characterization of gut bacteria in *B. tibialis* larvae parasitizing different developmental stages (3rd, 4th, and 5th instar) of the host (*A. pernyi* larvae). Results show that *B. tibialis* larval gut bacterial species were highly abundant. Significant differences in gut bacteria richness were observed among *B. tibialis* larvae parasitizing different host developmental stages, with ACE and Chao1 indices showing BT3 > BT4 > BT5 (*p* < 0.05), whereas the relative dominant bacteria structures were similar across groups. This may be associated with differences in nutritional metabolism and immune regulation across host developmental stages. Although different host developmental stages (*A. pernyi* larvae) may alter the gut bacterial composition of *B. tibialis* larvae, a functionally stable community structure is consistently maintained, suggesting that host physiological changes and dynamic shifts in the internal microenvironment may be key drivers of gut bacteria changes in *B. tibialis* larvae. PICRUSt2-based functional prediction indicates that the gut bacteria may play an important role during the parasitoid process. This study provides a new perspective for developing green biological control strategies against *A. pernyi*, offers important data for understanding the gut bacteria of tachinid flies (Diptera: Tachinidae), and lays a foundation for future in-depth research on the gut microbiota of host–parasitoid systems.

## Figures and Tables

**Table 1:** Basic information from sequencing and annotation of gut bacteria in *B. tibialis* larvae.

Samples		ASVs	Phylum	Class	Order	Family	Genus
BT3	1	153	12	21	41	57	83
	2	163	17	25	41	59	79
	3	122	12	19	36	48	67
	Total	344	23	36	70	105	157
BT4	1	56	4	7	16	23	27
	2	55	5	8	17	21	27
	3	57	7	10	19	26	30
	Total	138	11	16	33	45	58
BT5	1	29	5	8	13	19	21
	2	47	3	6	15	23	26
	3	26	4	7	12	17	18
	Total	76	6	10	24	36	43

Note: BT3 (1, 2, 3), three replicates of 3rd-instar *B. tibialis* larvae parasitizing 3rd-instar host *A. pernyi* larvae; BT4 (1, 2, 3), three replicates of 3rd-instar *B. tibialis* larvae parasitizing 4th-instar host *A. pernyi* larvae; BT5 (1, 2, 3), three replicates of 3rd-instar *B. tibialis* larvae parasitizing 5th-instar host *A. pernyi* larvae.

**Table 2:** Alpha diversity indices of gut bacteria in *B. tibialis* larvae.

Sample	Sobs	ACE	Chao1	Shannon	Simpson	Coverage
BT3	146.00 ± 21.38	148.82 ± 23.10	149.37 ± 24.10	3.66 ± 0.22	0.05 ± 0.01	0.998 ± 0.00
BT4	56.00 ± 1.00	56.46 ± 1.74	56.17 ± 1.26	3.22 ± 0.15	0.06 ± 0.01	0.999 ± 0.00
BT5	34.00 ± 11.56	34.12 ± 11.56	34.00 ± 11.36	2.82 ± 0.35	0.09 ± 0.03	0.999 ± 0.00

Note: Data were mean ± SD. BT3, 3rd-instar *B. tibialis* larvae parasitizing 3rd-instar host *A. pernyi* larvae; BT4, 3rd-instar *B. tibialis* larvae parasitizing 4th-instar host *A. pernyi* larvae; BT5, 3rd-instar *B. tibialis* larvae parasitizing 5th-instar host *A. pernyi* larvae.

## Data Availability

All metadata presented in this study is deposited in BioProject: PRJNA1452925. For further inquiries, please contact the first author.
